# VNTR-DAT1 and COMTVal158Met Genotypes Modulate Mental Flexibility and Adaptive Behavior Skills in Down Syndrome

**DOI:** 10.3389/fnbeh.2016.00193

**Published:** 2016-10-17

**Authors:** Laura del Hoyo, Laura Xicota, Klaus Langohr, Gonzalo Sánchez-Benavides, Susana de Sola, Aida Cuenca-Royo, Joan Rodriguez, Jose Rodríguez-Morató, Magí Farré, Mara Dierssen, Rafael de la Torre, The TESDAD Study Group, Aida Cuenca-Royo

**Affiliations:** ^1^Integrative Pharmacology and Systems Neuroscience Group, Neurosciences Research Program, IMIM-Hospital del Mar Medical Research InstituteBarcelona, Spain; ^2^Departament de Farmacologia, de Terapèutica i de Toxicologia, Universidad Autónoma de BarcelonaBarcelona, Spain; ^3^Department of Statistics and Operations Research, Universidad Politècnica de Cataluña/BarcelonaTechBarcelona, Spain; ^4^Cellular & Systems Neurobiology, Systems Biology Program, Centre for Genomic Regulation, The Barcelona Institute of Science and TechnologyBarcelona, Spain; ^5^Department of Experimental and Health Sciences, Universidad Pompeu FabraBarcelona, Spain; ^6^CIBER Fisiopatología Obesidad y Nutrición, Instituto Salud Carlos IIIMadrid, Spain; ^7^CIBER de Enfermedades Raras, Instituto Salud Carlos IIIMadrid, Spain

**Keywords:** Down syndrome, PFC-dependent cognition, dopamine, *COMTVal158Met*, *VNTR-DAT1*

## Abstract

Down syndrome (DS) is an aneuploidy syndrome that is caused by trisomy for human chromosome 21 resulting in a characteristic cognitive and behavioral phenotype, which includes executive functioning and adaptive behavior difficulties possibly due to prefrontal cortex (PFC) deficits. DS also present a high risk for early onset of Alzheimer Disease-like dementia. The dopamine (DA) system plays a neuromodulatory role in the activity of the PFC. Several studies have implicated trait differences in DA signaling on executive functioning based on genetic polymorphisms in the genes encoding for the catechol-O-methyltransferase (*COMTVal158Met)* and the dopamine transporter *(VNTR-DAT1)*. Since it is known that the phenotypic consequences of genetic variants are modulated by the genetic background in which they occur, we here explore whether these polymorphisms variants interact with the trisomic genetic background to influence gene expression, and how this in turn mediates DS phenotype variability regarding PFC cognition. We genotyped 69 young adults of both genders with DS, and found that *VNTR-DAT1* was in Hardy-Weinberg equilibrium but *COMTVal158Met* had a reduced frequency of *Met* allele homozygotes. In our population, genotypes conferring higher DA availability, such as *Met* allele *carriers* and VNTR-DAT1 *10-repeat* allele homozygotes, resulted in improved performance in executive function tasks that require mental flexibility. *Met* allele carriers showed worse adaptive social skills and self-direction, and increased scores in the social subscale of the Dementia Questionnaire for People with Intellectual Disabilities than *Val* allele homozygotes. The VNTR-DAT1 was not involved in adaptive behavior or early dementia symptoms. Our results suggest that genetic variants of *COMTVal158Met* and *VNTR-DAT1* may contribute to PFC-dependent cognition, while only *COMTVal158Met* is involved in behavioral phenotypes of DS, similar to euploid population.

## Introduction

Down syndrome (DS) is an aneuploidy syndrome that is most commonly caused by trisomy for human chromosome 21 (HSA21) leading to a cognitive and behavioral phenotype characterized by psychomotor delay, cognitive and behavioral deficits, and high risk for early onset of Alzheimer Disease (AD)-like dementia (Grieco et al., 2015). Executive function and adaptive behavior are impaired in DS adults, with the majority of studies showing specific deficits in working memory, attention, mental flexibility, and inhibitory control (Edgin et al., 2012). These functions are commonly ascribed to the prefrontal cortex (PFC) and frontostriatal networks. The function of these circuits relies heavily on neuromodulation, in particular on dopamine (DA) (Miller and Cohen, 2001) These findings are consistent with structural imaging evidences showing reductions in gray matter volumes in the frontal cortex and cingulate gyrus of DS adults (Rowe et al., 2006). While it is assumed that many of this phenotypic DS features stem from enhanced expression of a set of genes harbored within the HSA21, (Korenberg et al., 1994) there is few information about how genetic variants out of the chromosome 21 would be affected by the aneuploidy genomic context.

In the general population, polymorphisms known to influence DA levels in the PFC, such as *VNTR* in the gene of the DA transporter (DAT) and *Val158Met* in the gene of the catechol-O-methyltransferase (COMT), are associated to PFC executive function (Caldú et al., 2007; Witte and Flöel, 2012). The *COMTVal158Met* polymorphism (rs4680) is a SNP (single nucleotide polymorphism) that causes the substitution of a Valine for a Methionine at position 158/108 (Lachman et al., 1996) and has been shown to affect COMT expression, which is reduced in the *Met* allelic variant carriers. The *DAT1* polymorphism is a *VNTR* (variable number tandem repeat) of 40 nucleotides that can range from three to eleven repetitions and has been shown to affect expression of DAT, with the *10-repeat* allele being associated with lower DAT levels than the *9-repeat* allele (van de Giessen et al., 2009; Shumay et al., 2011). Both the *Met* allelic variant and *10-repeat* allele would result in greater availability of DA in the PFC, which may be linked to better PFC-dependent cognitive skills (Meyer-Lindenberg et al., 2005; Wonodi et al., 2009; Barnett et al., 2011), whereas these same alleles had been associated with a disadvantage in adaptive behavior (Mier et al., 2009; Koutsilieri et al., 2014).

In DS human brains, the response to dopaminergic stimulation is depressed in post-mortem cerebral cortex (Lumbreras et al., 2006), and reductions in dopamine and its acidic metabolites (DOPAC and HVA) have been detected in DS fetal and adult brains (Risser et al., 1997; Schneider et al., 1997; Whittle et al., 2007). However, only two studies have explored DA genetic polymorphisms. A family-based analysis revealed significant over-transmission of a DRD4 VNTR allele, which encodes for D4 dopamine receptor for DS (Das Bhowmik et al., 2008). In a second study, the *7-repeat* allele of the DRD4 polymorphism was associated to behavioral and executive functions difficulties in children with DS (Mason et al., 2015).

Since it is known that the phenotypic consequences of genetic variants are modulated by the genetic background in which they occur, we here explored whether *COMTVal158Met* and *VNTR-DAT1* variants interact with the trisomic genetic background to influence gene expression, and how this in turn mediates DS phenotypes. We hypothesized that *COMTVal158Met* and *VNTR-DAT1* polymorphisms may influence cognitive and behavioral skills differentially to euploid population.

## Materials and Methods

### Subjects

For this cross-sectional study 87 young adults of both genders with DS were drawn from the baseline exploration of a clinical trial carried out by our research group (TESDAD Study ClinicalTrials.gov Identifier: NCT01699711). Participants were excluded if they had neurological disorders other than DS, relevant medical diseases, co-morbid mental disorders or were under any pharmacological treatment that could interfere with cognitive function. Exclusion criteria also included severe language deficit (significant speech and/or comprehension limitations), behavioral disturbances and/or poor level of collaboration during the assessment. Four subjects were excluded for speech and language comprehension limitations, and two declined to participate. Subject disposition, withdrawals and the composition of the primary analysis population are shown in the CONSORT diagram (Supplementary Figure 1).

In the present study, the genotype analysis was performed in the whole eligible sample (*n* = 81), 10 subjects could not be genotyped because an insufficient amount of DNA and 11*–repeat* allele carriers were excluded of the DAT polymorphism analysis since we only detected two subjects, resulting in a final sample of 69 participants of both genders (*M* = 35, *F* = 34) with any of the DS genetic variations (trisomy 21; *n* = 65, partial trisomy; *n* = 2, mosaic; *n* = 1 or translocation; *n* = 1), mean age of 25 years (range = 18–37, *SD* = 4.4) and mean IQ of 45 (range = 40–86, *SD* = 8.7).

The data of a sample of healthy control Spanish subjects genotyped for *COMTVal158Met* [*n* = 93, mean age = 22.8 (4.1), sex = 49 M, 44 F; (Cuyàs et al., 2011)], and *VNTR-DAT1* [*n* = 57, mean age = 22.7 (2.83), sex = 57 M, 0 F; unpublished data] were drawn from a previous study (CEIC-IMAS 99/935/I 2001/1226/I), and were used to assess whether the genotype frequency in the TESDAD DS population was in equilibrium.

### Genotype Analysis

Genomic DNA was extracted from the peripheral blood leukocytes of all the participants using Flexi Gene DNA kit (Qiagen Iberia, S.L., Spain) according to the manufacturer instructions. *COMTVal158Met* allelic variants were determined as previously described (Verdejo-García et al., 2013). VNTR polymorphism of VNTR-DAT1 was evaluated by PCR using the following primers 5′TGTGGTGTAGGGAACGGCCTGAG-3′ (forward) and 5′CTTCCTGGAGGTCACGGCTCAAGG-3′ (reverse), the reaction conditions for this experiment were the following: 1X PCR Amplification buffer and 1X PCR Enhancer solution (Invitrogen, Carlsbad, CA), 1.5 mM MgSO4, 0.2 mM dNTPs, 0.2 μmol of each primer, 1.75 U of Taq DNA polymerase (Invitrogen), and approximately 50 ng of genomic DNA as template. The reaction was carried out on the following conditions: an initial denaturation of 5 min. at 95°C, followed by 50 cycles as follows: 30 s denaturation at 95°C, 30 s annealing at 63°C, and 45 s elongation at 68°C, and a final elongation step at 72°for 5 min. The PCR products were separated by electrophoresis on a 2% agarose gel, being the 9-allele 440 bp, and the 10-allele 480 bp.

### Neuropsychological Testing

We focused on PFC-dependent cognition, exploring components of executive functioning, specifically the following domains: impulsivity, attention, working memory, mental flexibility, planning ability, inhibitory control and adaptive behavior. A detailed description of the tests is provided in Supplementary Annex 1.

Intellectual quotient (IQ) was estimated using the Kaufman Brief Intelligence Test (K-BIT). Impulsivity was measured analyzing the relation between response times and errors in a set of tests from the non-verbal Cambridge Neuropsychological Test Automated Battery (CANTAB): Motor screening test (MOT), Simple Reaction Time (SRT), and Spatial Span Forward Recall (SSP-FR). To assess the attentional span the Digits Span Forward Recall (DSP-FR) from WAIS-III and the Spatial Span Forward (CANTAB) were administered. Working memory for visual and verbal information was evaluated with the Spatial Span Backward Recall (SSP-BR, CANTAB) and the Digits Span Backward Recall (DSP-BR) from WAIS-III, respectively. Mental flexibility was measured rating switching ability and reversal learning. Switching ability (times subject change from one semantic category to a new one) was assessed with the Semantic Fluency Word Generation Task (SFWGT, animals in 1 min), and reversal learning (disengagement from inappropriate strategies when reinforcement contingencies change), with the Weigl Color-Form Sort Test (WCFST). Planning ability was measured using the Tower of London from Drexel University (ToLDx) and the Cats & Dogs test was used to assess inhibitory control. To assess adaptive behavior, the adult version of the Adaptive Behavior Assessment System-Second Edition (ABAS-II) was used.

Finally, since early Alzheimer’s disease like dementia in DS population courses with a progressive decline of the DA system and a faster degeneration of the PFC, affecting executive function (Fernandez and Reeves, 2015), we explored early dementia symptoms with the Dementia Questionnaire for People with Intellectual Disabilities (DMR).

### Statistical Analysis

Descriptive analyses were carried out for sociodemographic and clinical parameters of all the participants and for all neuropsychological variables, providing measures of mean and standard deviation.

The genotypic frequency of the different polymorphisms was tested by Hardy-Weinberg equilibrium. To study the association between polymorphisms and cognition, two-way ANOVA models were fitted for each of the cognitive tests and questionnaires scores. The 2 genes were included in the models as independent factors and treated as binary variables, *Val* allele homozygotes vs. *Met* allele carriers (*COMTVal158Met*) and *10-repeat* allele homozygotes vs. *9-repeat* allele carriers (*VNTR-DAT1*), respectively. In addition, possible sources of epistatic effects on cognition were considered by assessing the interaction between *COMTVal158Met* and *VNTR-DAT1* genotypes in each of the two-way ANOVA models.

The statistically significant associations were fixed at a significance level of 0.05, using the model-based estimated mean differences as the effect size of interest. All statistical analyses were performed using the statistical software packages SPSS (Version 18.0; SPSS Inc., Chicago, IL, USA) and R (Version 3.1.1; The R Foundation for Statistical Computing, Vienna, Austria).

## Results

### Distribution of Polymorphisms

Socio-demographic data and cognitive parameters for all the neuropsychological variables of the 69 participants are provided in the Supplementary Table 1.

In our study, the euploid Spanish population was used to obtain the expected frequencies for the Hardy-Weinberg equilibrium (**Table 1**). The proportion of *COMTVal158Met* allelic frequencies among the Spanish reference population (*n* = 93) was similar to the reference CEU (Utah residents with Northern and Western European ancestry), and TSI (Tuscans from Italy) HapMap populations (HapMap, n.d.); (**Table 1**). No data from CEU and TSI HapMap reference populations was available for the proportion of VNTR-DAT1 allelic frequencies.

**Table 1:** Distribution of *COMTVal158Met* and *VNTR-DAT1* allele frequencies in the DS and reference populations.

		Hap map CEU population	Hap map TSI population	Spanish population	TESDAD DS population (Spanish)
COMTVal158Met					
	Met/Met (A/A)	28 (0.248)	20 (0.196)	21 (0.225)	6 (0.087)
	Val/Met (G/A)	52 (0.460)	52 (0.510)	45 (0.484)	38 (0.550)
	Val/Val (G/G)	33 (0.292)	30 (0.294)	27 (0.290)	25 (0.362)
VNTR-DAT1	10/10			30 (0.526)	35 (0.507)
	10/9			23 (0.403)	26 (0.377)
	9/9			4 (0.070)	8 (0.116)

*CEU, Utah residents with Northern and Western European ancestry; the TSI, Tuscans from Italy; DS, Down syndrome. Data represent the number of individuals bearing each genotype, and parenthesis account for genotypic frequencies (0–1).*

In the TESDAD DS population, we found a Hardy–Weinberg disequilibrium (χ2 = 6.47, *p* < 0.039) due to an under-representation of the *Met/Met* genotype in the *COMTVal158Met* polymorphism, as compared to the CEU, TSI, and Spanish populations. Conversely, *VNTR-DAT1* allele frequency was in the expected Hardy–Weinberg equilibrium compared to the Spanish population (*n* = 57); (**Table 1**).

### *VNTR-DAT1* and *COMTVal158Met* Effects on Cognitive and Functional Performance

**Table 2**; **Figures 1** and **2** show the main effects in components of executive functioning and caregiver’s rating scores between *VNTR-DAT1* and *COMTVal158Met* genotypes. The VNTR-DAT1 *10-repeat* allele homozygotes showed statistically significant shorter response times in the CANTAB Simple Reaction Time and Spatial Span Forward Recall tasks, not linked to impulsivity since they were not accompanied by a higher number of errors. *10-repeat* allele homozygotes also showed statistically significant better performance of tasks requiring mental flexibility, as measured by the SFWGT, but not by the WCFST. Taken together, these data indicate that in our DS population the *10-repeat* allele homozygotes are faster responders in attentional and psychomotor tasks and have better mental flexibility. No statistically significant association was found between the *VNTR-DAT1* polymorphism and parental reports (ABAS-II and DMR).

**Table 2:** *VNTR-DAT1* and *COMTVal158Met* effects on cognitive and functional variables in the TESDAD DS population.

Test	Function assessed	Polymorphism	Figure	Estimate	Standard error	*t*-value	*p*-value
SSP-FR (CANTAB)	Mean time to response	VNTR-DAT	**Figure 1A**	537	270	1.999	**0.05**
SRT (CANTAB)	Mean time to respond	VNTR-DAT	**Figure 1B**	117	48	2.424	**0.018**
MOT(CANTAB)	Mean time to respond	VNTR-DAT		137	77	1.772	0.081
SFWGT	Switching ability: MF	VNTR-DAT	**Figure 1C**	–1.5	0.5	–3.265	**0.002**
WCFST	Reversal learning: MF	VNTR-DAT		–0.8	0.4	–1.921	0.059
SFWGT	Switching ability: MF	COMTVal158Met	**Figure 2A**	–1.4	0.5	–2.935	**0.005**
ABAS-Social Skills	Adaptive social behavior	COMTVal158Met	**Figure 2B**	6.99	2.9	2.453	**0.017**
ABAS-Self-Direction	Capacity of regulate behavior to the demands of a situation.	COMTVal158Met	**Figure 2C**	7.65	3.7	2.08	**0.041**
DMR-SOS	Social symptoms of dementia	COMTVal158Met	**Figure 2D**	–2.1	1	–2.108	**0.039**

*SSP, Spatial Span; FR, Forward Recall; SRT, Simple Reaction Time; MOT, Motor Screening; SFWGT, Semantic Fluency Word Generation Task; WCFST, Weigl Color-Form Sort Test; CANTAB, Cambridge Neuropsychological Test Automated Battery; DMR-SOS, Dementia Questionnaire for Persons with Intellectual Disabilities (Sum of Social Scores); ABAS, Adaptive Behavior Assessment System; MF, mental flexibility; Estimate, estimated mean difference; Standard Error, standard error of the estimated mean difference; *t*-value, Value of the *t*-statistics; F, Figure; G, Graphic. Two-way ANOVA model was carried out to analyse main differences between allelic groups of each polymorphism regarding cognitive and functional outcomes.*

*Statistically significant associations above a significance level of 0.05 are represented as bold values.*

**FIGURE 1:**
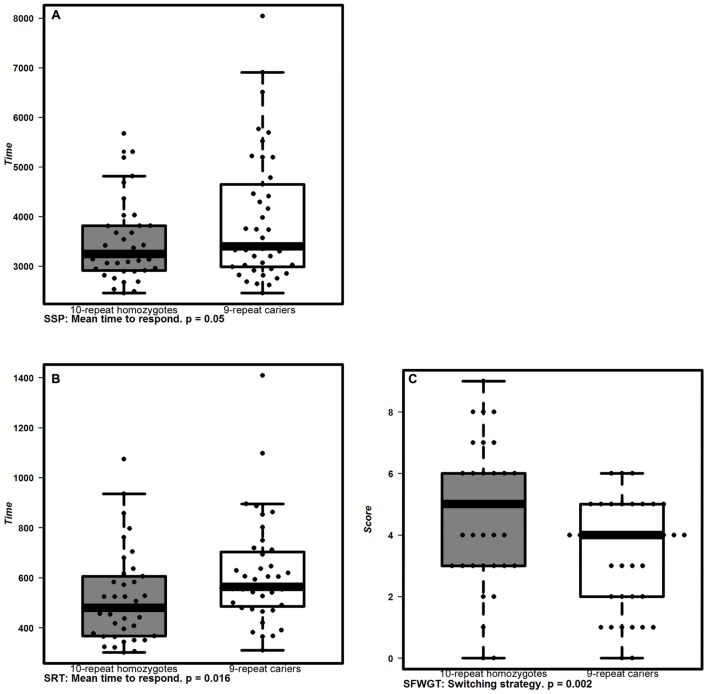
**Cognitive performance associated with *VNTR-DAT1* polymorphisms.** Box plots represent the cognitive performance of allelic groups of *VNTR-DAT1* polymorphism. 10-repeat homozygotes (gray boxes) displayed shorter response times in CANTAB **(A)** SSP (*p* < 0.05; two way ANOVA) and **(B)** SRT tasks (*p* < 0.016; two way ANOVA), and better mental flexibility measured by **(C)** SFWGT (Switching strategy; *p* < 0.016; two way ANOVA) than 9-repeat carriers (white boxes). The boxplots show the median and interquartile range. Dots indicate individual values.

**FIGURE 2:**
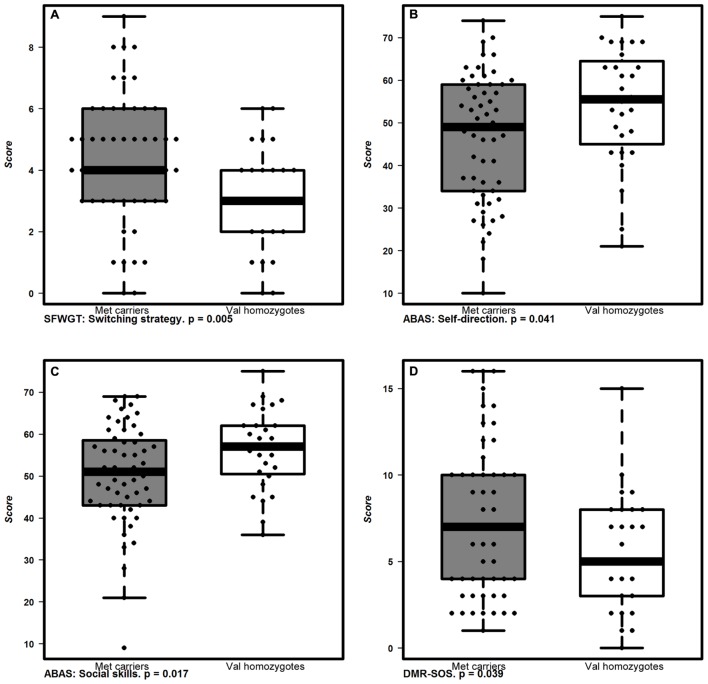
**Cognitive and functional performance associated to *COMTVal158Met* polymorphisms.** Box plots represent the behavioral and cognitive performance of allelic groups of *COMTVal158Met* polymorphism. *Met* carriers (gray boxes) performed better in mental flexibility measured by **(A)** SFWGT (Switching strategy; *p* < 0.005; two way ANOVA) and had worse parental reports in two ABAS-II subscales: **(B)** ABAS-Self-Direction (*p* < 0.041; two way ANOVA) and **(C)** ABAS-Social Skills (*p* < 0.017; two way ANOVA), and also in the **(D)** social scale of the DMR; DMR-SOS (*p* < 0.039; two way ANOVA) than *Val* homozygotes (white boxes). The boxplots show the median as well as the interquartile range. Dots indicate individual values.

Regarding *COMTVal158Met, Met* allele carriers showed no differences in response time in the CANTAB tasks but we detected the same profile as for *10-repeat* allele homozygotes regarding mental flexibility that was better as assessed by the SFWGT, but not WCFST. In addition, *Met* allele carriers had statistically significant worse parental reports referred to adaptive behavior, specifically in the social skills and self-direction subscales of ABAS-II, and statistically significant higher early dementia rates in the DMR social scale (DMR-SOS) than *Val* allele homozygotes. Thus, in our population, the *Met* allele carriers have a greater mental flexibility but worse adaptive behavior, along with higher social deterioration.

No statistically significant differences were found between *VNTR-DAT1* or *COMTVal158Met* genotypes with regards to attentional span (The Digits Span Forward Recall, The Spatial Span Forward Recall from CANTAB), working memory (the Digits Span Backward Recall from WAIS and the Spatial Span Backward Recall from CANTAB), planning ability (the Tower of London from Drexel University, ToLDx), or inhibitory control (the Cats & Dogs test).

Interactions between genotypes were considered for each cognitive and functional variable; however, no statistically significant genotype interaction between *VNTR-DAT1* and *COMTVal158Met* polymorphisms was detected.

## Discussion

Since it is known that the phenotypic consequences of genetic variants are modulated by the genetic background in which they occur, we here examined the association between DA related polymorphisms *VNTR-DAT1* and *COMTVal158Met* and inter-individual differences in executive function, adaptive behavior and early symptoms of dementia in DS young adults. We show that genotypes conferring higher DA synaptic availability (*Met* allele carriers and *10-repeat* allele homozygotes), result in improved performance in executive function tasks that require mental flexibility. We also observe worse social skills and self-direction and earlier social symptoms of dementia in *Met* allele carriers but not in *10-repeat* allele homozygotes.

In our DS population the genotype distribution for *VNTR-DAT1* was in Hardy-Weinberg equilibrium, but the *COMTVal158Met* had a reduced frequency for *Met* allele homozygotes. Even though, there is a need of a more extensive analysis of the frequencies of the *COMTVal158Met* polymorphism in the DS population, and a triplet analysis would be required to confirm this observation, the reduced *Met* frequency in our population could be explained as a case of natural selection against the *Met* allele homozygotes since in the general population it has been observed that women who are carriers of the *Met* allele have a higher risk of miscarriage (Khadzhieva et al., 2014).

We here hypothesized that DA related polymorphisms would impact executive function in DS, but not necessarily in the same way as in euploid population, since the genetic trisomic background and the underlying cognitive deficits may influence the variants’ effects. Main differences in cognitive performance between DS and age-matched controls have already been reported in our previous works (de Sola et al., 2015; Hoyo et al., 2015). Specifically, we detected a substantial deficit on executive functions, being the most impaired: semantic verbal fluency (SFWGT: total word production) and mental flexibility (SFWGT: switching ability). In our DS group, the switching ability, which allow subjects to change from one semantic category to another, is associated (*p* < 0.001, *r* = 0.689) to a higher word production leading to a better performance of the task.

We here found that *10-repeat* allele homozygotes were faster respondents in attentional tasks (SRT and SSP-FR), and this same genotype and *Met* allele carriers were associated to better mental flexibility showing better switching abilities (SFWGT).

Previous studies reported that healthy subjects carrying the *10-repeat* allele were more impulsive than *9-repeat* allele carriers, being faster but committing more errors in a non-verbal attentional task (CPT, CANTAB; Caldú et al., 2007). Here, the faster responses of *10-repeat* allele homozygotes as compared to the *9-repeat* carriers could not be considered as a sign of impulsivity as there was no association between faster responses and the errors made. An association between *10-repeat* allele and ADHD (Cornish et al., 2005), in which impulsivity is a core characteristic (Winstanley et al., 2006), has also been demonstrated, being the hyperactive/impulsive-ADHD-type linked with the 10-*repeat* allele (Waldman et al., 1998). Although there is a high ADHD prevalence in DS population (Ekstein et al., 2011), in our study there was no ADHD comorbidity nor signs of impulsivity at a group level, so we could not determine such association.

The association between *Met* allele carriers and better mental flexibility is in agreement with previous studies in general population in which subjects with the low-activity *Met* allele were associated to fewer perseverative errors in a mental flexibility task (Malhotra et al., 2002).

Taken together, our results indicate that *10-repeat* allele homozygotes and *Met* allele carriers in our DS population have better mental flexibility. Interestingly, a reduction of the levels of DA in the frontal cortex of fetal DS brains has been observed (Whittle et al., 2007) that is associated with dysfunctional neuronal development in DS. Therefore, even though we have not directly measured PFC DA availability, *Met* allele carriers and *10-repeat* allele homozygotes, which are supposed to have higher frontal DA availability, could be compensating PFC dependent executive functions (Rowe et al., 2006).

Since executive skills are strongly associated with adaptive behavior (ABAS-II), we explored how these polymorphisms were linked to adaptive behavior skills. Our results showed differences in parental-reports between *COMTVal158Met* genotypes but not between *VNTR-DAT1* genotypes. *Met* allele carriers showed worse adaptive behavior, concretely worse social skills and self-direction. Self-direction is a subscale of the ABAS-II which measures the executive control in daily living. Social abilities are also mediated by executive function, which helps the subject to adapt to different social situations regulating emotion (Campbell et al., 2015). Previous studies in general population have also find a positive association between the *Val* allele and social skills (Walter et al., 2011). Our results showed that the *Met* allele is conferring an advantage in cognition (mental flexibility) but penalizing for adaptive behavior skills, which has been shown either in healthy and disease conditions (Mier et al., 2009).

Finally, *Met* allele carriers were also associated to worse scores in the social scale of the DMR (DMR-SOS) than high-activity *Val* allele homozygotes. Conversely, some reports suggest that increased synaptic DA catabolism promotes the neurodegeneration within DA-innervated brain regions and thus, progressive cognitive and behavioral decline associated with AD (Gennatas et al., 2012). However, even though the *Met* allele (increasing DA availability) may confer resistance to AD (Gennatas et al., 2012), in our sample *Met* allele carriers did not show differences in the general DMR score. Thus, we interpret this result as an association between *Met* allele and social deterioration (DMR-SOS), instead of a link with early social dementia symptoms, since *Met* allele carriers also showed worse adaptive social behavior (ABAS-II).

In DS, one study has reported that the DRD4 *7-repeat* allele was related to behavioral and executive functions difficulties (Mason et al., 2015). We here did not explore these variants and thus, possible interactions of our polymorphisms and the *DRD4* gene variants could also be contributing to the detected phenotypes. Other genotypes examined seem to little contribute (genetic polymorphisms in the dopaminergic and serotonergic system the DRD4 exon 3 VNTR and the 5-HTTLPR) to the cognitive dysfunctions observed in DS (Das Bhowmik et al., 2008).

### Limitations

First, even though the sample size of this study is larger than other single-gene association studies in people with DS (Mason et al., 2015), a larger sample size would have been ideal to confirm our results. However, limited sample sizes are expected in most studies conducted in special populations. Secondly, we did not have a comparison group, although our effects were similar to typically developing samples reported in the literature. Future studies should explore whether *COMTVal158Met* and *VNTR-DAT1* effects on executive function and behavior in adults with DS are additive or enhanced relative to effects in typically developing individuals. Finally, the large number of statistical tests carried out may increase the probability of Type-1 errors. Nonetheless, since this is rather an exploratory than a confirmatory study, no correction to control a family-wise significance level of 0.05 has been applied in order not to increase the probability of Type-2.

## Conclusion

Our study has evaluated whether allelic variants of specific non-trisomy 21 genes (*COMTVal158Met* and *VNTR-DAT1*) could contribute to explain cognitive and behavioral variability in DS phenotype. While the phenotype is most likely due to a subtle increase in gene dosage, how genetic variants out of the chromosome 21 and related to DA transmission would affect variability in prefrontal cognition in an aneuploidy genomic context is little explored. Since it is known that the phenotypic consequences of genetic variants are modulated by the genetic background in which they occur, we here explore whether *COMTVal158Met* and *VNTR-DAT1* variants interact with the trisomic genetic background to influence gene expression, and how this in turn mediates Down syndrome phenotypes. Our results suggest that genotypes conferring higher DA availability as for *Met* allele carriers and *10-repeat* allele homozygotes result in improved mental flexibility. With respect to parental reports, worse rates in adaptive behavior regarding social skills and self-direction were found only in the *Met* allele carriers, along with higher social deterioration. We conclude that genetic variations related to DA transmission may have an impact on the cognitive and behavioral DS phenotype similar to the general population. Nevertheless, further longitudinal studies are needed to confirm our results and determine whether these differences are unaffected or significantly enhanced by Trisomy 21 on molecular (epigenetic) and cognitive/behavioral and functional levels.

## Ethical Standards

The authors assert that all procedures contributing to this work comply with the ethical standards of the relevant national and institutional committees on human experimentation and with the Helsinki Declaration of 1975, as revised in 2008. The study was conducted in accordance with the Declaration of Helsinki and Spanish laws concerning data privacy. The study was conducted in accordance with the Declaration of Helsinki and Spanish laws concerning data privacy. The protocol was approved by the Ethical Committee of the Parc de Salut Mar of Barcelona (CEIC-PSMAR). Upon arrival at the research centre (Hospital del Mar Medical Research Institute-IMIM), participants, parents and legal guardians (in case of legal incapacitation) were informed of the ensuing protocol and they gave their written informed consent before participating.

## Members of the Tesdad Study Group

**Aida Cuenca-Royo**, Hospital del Mar Medical Research Institute, Barcelona, Spain, Neuropsychologist, Site Investigator; **Alessandro Principe**, Hospital del Mar Medical Research Institute, Barcelona, Spain, Neurophysiology Section, Site Investigator; **Bessy Benejam**, Catalan Foundation of Down Syndrome, FCSD, Barcelona, Spain, Neuropsychologist, site investigator; **Ester Civit**, Hospital del Mar Medical Research Institute, Barcelona, Spain, Site Investigator; **Gimena Hernandez**, Hospital del Mar Medical Research Institute, Barcelona, Spain, Site Investigator; **Gonzalo Sánchez-Benavides**, Hospital del Mar Medical Research Institute, Barcelona, Spain, Neuropsychologist, Site Investigator; **Henri Bléhaut**, Jérôme Lejeune Foundation, Paris, France, Site investigator; **Iván Dueñas**, Hospital del Mar Medical Research Institute, Barcelona, Spain; **Jesús Pujol**, Neurovoxel, Barcelona, Spain, neurologist/neuroimaging, Site Investigator; **Joan Rodríguez**, Hospital del Mar Medical Research Institute, Barcelona, Spain, Study Coordinator; **Jordi Peña-Casanova**, Hospital del Mar Medical Research Institute, Barcelona, Spain, Dementia Section, Site Investigator; **Josep M**^a^ **Espadaler**, Hospital del Mar Medical Research Institute, Barcelona, Spain, Neurophysiology Section, Site Investigator; **Judit Sánchez**, Neuropsychlogist, Feskits, Barcelona, Spain, Site Investigator; **Katy Trias**, Catalan Foundation of Down Syndrome, FCSD, Barcelona, Spain, Site Investigator; **Klaus Langohr**, Polytechnics University, Barcelona, Spain, Statistician; **Laia Roca**, Hospital del Mar Medical Research Institute, Barcelona, Spain, Study Manager; **Laura Blanco**, Hospital del Mar Medical Research Institute, Barcelona, Spain, Neuropsychologist, Site Investigator; **Laura del Hoyo**, Hospital del Mar Medical Research Institute, Barcelona, Spain, Neuropsychologist, Site Investigator; **Laura Xicota**, Hospital del Mar Medical Research Institute, Barcelona, Spain, Site Investigator; **Magí Farr**, Hospital del Mar Medical Research Institute, Barcelona, Spain, co-PI; **Mara Dierssen**, Centre for Genomic Regulation- CRG of Barcelona, co-PI; **Rafael de la Torre**, Hospital del Mar Medical Research Institute, Barcelona, Spain, PI; **Rut Freixas**, Centre for Genomic Regulation- CRG of Barcelona, Site Investigator; **Sebastiá Videla**, Catalan Foundation of Down Syndrome, FCSD, Barcelona, Spain, site investigator; **Silvina Catuara-Solarz**, Centre for Genomic Regulation- CRG of Barcelona, Site Investigator; **Susana de Sola**, Hospital del Mar Medical Research Institute, Barcelona, Spain, Neuropsychologist, Site Investigator; **Valérie Legout**, Jérôme Lejeune Foundation, Paris, France, Site Investigator.

## Author Contributions

LdH conceived and design this study, carried out the neuropsychological assessment, collected and analyzed the data, interpreted the results, and wrote the manuscript. LX and JR-M carried out the genotyping; LX interpreted the results, and contributes to manuscript preparation. KL reviewed and carried the statistical analysis, and manuscript preparation. SS, AC-R, and GS-B carried out the neuropsychological assessment. LdH and JR carried out the medical explorations, and coordinate the experiments. MF, MD, and RT conceived the study design and reviewed the manuscript. All authors read and approved the manuscript.

## Conflict of Interest Statement

The authors declare that the research was conducted in the absence of any commercial or financial relationships that could be construed as a potential conflict of interest.

The reviewer DM and handling Editor declared their shared affiliation, and the handling Editor states that the process nevertheless met the standards of a fair and objective review.
