# Supplementary material for: VNTR-DAT1 and COMTVal158Met Genotypes Modulate Mental Flexibility and Adaptive Behavior Skills in Down Syndrome
Source: Front Behav Neurosci. 2016 Oct 17;10:193. doi: 10.3389/fnbeh.2016.00193 (PMC5065956; doi:10.3389/fnbeh.2016.00193)
Supplement: Supplementary file 2 [file Data_Sheet_1.DOCX]

**Supplementary Figure 1.** CONSORT diagram showing the flow of participants (subject disposition, withdrawals and the composition of the primary analysis population).

Assessed for eligibility (n=87)

Excluded (n=81)

- Not meeting inclusion criteria for the administered tests: speech and comprehension limitations (n = 4)
- Declined to participate (n = 2)

Analysed (n = 69)

- Excluded from analysis if they were 11-repeat allele carriers (n = 2)

**Analysis**

Genotype analysis (n=81)

**Enrollment**

Excluded (n = 71)

- Not successfully genotyped (n = 10)
