# Supplementary material for: VNTR-DAT1 and COMTVal158Met Genotypes Modulate Mental Flexibility and Adaptive Behavior Skills in Down Syndrome
Source: Front Behav Neurosci. 2016 Oct 17;10:193. doi: 10.3389/fnbeh.2016.00193 (PMC5065956; doi:10.3389/fnbeh.2016.00193)
Supplement: Supplementary file 3 [file Data_Sheet_2.DOC]

**Supplementary Annex 1. Neuropsychological assessment**

1. *Cognitive assessment and intellectual quotient estimation (IQ)*

*Intellectual quotient*

The Kaufman Brief Intelligence Test (K-BIT, Spanish version) (Kaufman & Kaufman, 1994), This tool meets the need to evaluate the intellectual status of individuals covering a wide age range, from 4 to 90 years, and significantly correlates with the WISC-III (Canivez, 2005). The intellectual quotient corresponds to the K-BIT standardized total score and was considered in the analyses to control for clinically relevant pre-existing differences in general intellectual disability among individuals.

*Psychomotor speed*

Motor Screening test (MOT, from the CANTAB) (Cognition, 1996) Participants are instructed to touch a series of crosses that appear randomly on the screen. This task assesses psychomotor speed and accuracy. The measure of response latency (in milliseconds) was considered in the present study.

*Attention*

Simple Reaction Time (SRT, from the CANTAB) (Cognition, 1996) In this test, subjects must press the button on a press pad as soon as they see a square appearing in the middle of the screen. Intervals between the examinee’s response and the onset of the next stimulus are variable during task performance. This task tests general alertness and motor speed. Measures of accuracy (number of correct trials; range: 0-100) and response latency (in milliseconds) were used for the analyses.

Digit Span: forward recall (from the WAIS-III, Spanish version; (Wechsler, 1997). Subjects are required to listen to a series of numbers with randomized presentation, and then repeat them back. The length of the series increases with the subject’s success. Forward recall score provides a good measure of verbal attention and memory span.

Spatial Span (SSP): forward recall (from the CANTAB)(Cognition, 1996) This test constitutes an analogue of the Corsi block task (Milner, 1971). Subjects must memorize and reproduce a sequence of spatial locations demonstrated on the computer screen. Participants view a sequence of squares that change, one at a time, from white to a different color. The examinee has to touch on the screen the squares in the same order as they were presented. Length of the sequence increases with the subject’s success. Forward recall is predominantly a measure of visual attention and memory span. Measures of span length and total number of errors were considered for the analyses.

*Executive functions*

Digits Span: backward recall (from the WAIS-III, Spanish version) (Wechsler, 1997). Subjects are required to listen to a series of numbers with randomized presentation, and then repeat them backwards. The length of the series increases with the subject’s success. Backward recall score is predominantly a measure of verbal working memory span.

Spatial Span (SSP): backward recall (from the CANTAB) (Cognition, 1996) Subjects are required to memorize and inversely reproduce a sequence of spatial locations that appear on the computer screen. The examinee has to reproduce this sequence backwards by touching items in the inverse order they were originally presented. Length of the sequence increases with the subject’s success. Backward recall is predominantly a measure of visual working memory span. Measures of span length and total number of errors were used for the analyses.

Semantic Fluency Word Generation Task, SFWGT (Benton, Hamsher, & Sivan, 1976) Subjects are asked to generate as many words as possible in 1 minute belonging to the specified category of “animals”. As in the previous test, high scores indicate greater verbal fluency ability. Switching and clustering were also codified. Mean cluster size was the main dependent variable for clustering, whereas number of switches was the main dependent variable for switching. A cluster was defined as any series of two or more successively produced words belonging to the same semantic subcategory. Cluster size was computed by adding up series of words from the same subcategory starting from the second word within each cluster (i.e. a three-word cluster has a size of two). The number of switches was defined and computed as the number of times the participant changed from one cluster to another. Two clusters may also be overlapping, for example, from “farm animals” to “birds” in “cow–pig–chicken–pigeon–eagle.” Here, one switch is made between the cluster “cow–pig–chicken” and “chicken–pigeon–eagle.” The computation of number of switches included single-word clusters. An inter-rater reliability analysis was performed and the reliability studied by means of the intra-class correlation coefficient (ICC) was high with values ranging from 0.89 to 0.98.

Tower of London-Drexel University, ToLDx (Culbertson & Zillmer, 2005) For this study we used the pediatric version (ages 7 to 15) in order to avoid floor effects. Standard norms were used from this version from the upper age range: 13 to 15 years old. This test requires the movement of three different colored balls across three different sized pegs in order to replicate a goal configuration. Movements follow strict rules. The first two problems were training tasks, following which 10 problems of increasing complexity were presented. The task ceased after the examinee failed to solve two consecutive problems. For those subjects who failed to complete the entire test, number of movements and problem-solving time were adjusted for the number of uncompleted trials to allow a feasible comparison with higher performers, assigning the maximum number of movements (20) and maximum problem-solving time (120 s) to each failed item. Scores for total problem-solving time (in seconds), total number of moves needed to complete the configuration, and total number of problems solved within the minimum number of moves were used for the analyses, providing a good measure of planning ability.

Weigl Color-Form Sort Test, WCFST (Goldstein, Scheerer, & Hanfmann, 1953) This is a set-shifting task that assesses the ability to categorize across two dimensions: color and shape. Instructions for administration and scoring were taken from Strauss & Lewin, 1982 (Strauss & Lewin, 1982)Test material consists of 12 tokens: four circles, four triangles, and four squares, shapes are colored blue, red, yellow or green. The 12 tokens are displayed unsorted in front of the examinee. In the first trial the examinee is required to sort the tokens in a way that they go together (color or shape). After this first trial, the examinee is required to sort them again but using a different combination. The examinee receives prompting from the examiner if he or she is not able to change the sorting principle used in the first trail. Scores range from 0 to 5. A maximum score of 5 points is obtained when the subject is able to shift the initial category without a prompt, whereas 0 points are obtained if the subject fails despite prompting. A higher score indicates greater capacity for set-shifting and is considered a good estimation of cognitive flexibility or reversal learning in individuals with intellectual disability.

Cats & Dogs Test (Ball, Holland, Treppner, Watson, & Huppert, 2008)This is a Stroop-like task assessing response inhibition, based on the original Day–Night task (Gerstadt, Hong, & Diamond, 1994). In this test, a sequence of 16 pictures, 8 cats and 8 dogs arranged in a prefixed order, are presented to the examinee on a single strip of card. The task consists of two trials with two different conditions: a control and an experimental-inhibition trial. In the control the examinee is required to point to each picture in turn and name the animal as quickly as possible. In the experimental-inhibition condition, the examinee is instructed to say ‘dog’ when he or she points to a cat and to say ‘cat’ when pointing to a dog. A practice trial is given to the examinee before the performance of both trial conditions. Measures of task accuracy in the experimental inhibition trial (total number of correct responses) and total time performance (in seconds) were included in the analyses.

1. *Functional assessment*

*Adaptive behavior in daily living*

The Adaptive Behavior Assessment System-Second Edition adult version, ABAS-II (Harrison & Oakland, 2003) ABAS-II was designed, according to AAMS guidelines, for evaluating adaptive skills in people with mental disabilities of a wide age range and across multiple environments. The ABAS-II tool for adults (ages 16 to 89) includes 5 subscales which assess the individual’s competence (in terms of behavior frequency) in 10 different skill areas: communication abilities, community use, functional academics, home living, health and safety, leisure, self-care, self-direction, social interaction, and working/labor skills. All answers to this questionnaire were self-reported by parents. Raw scores of the dimensions and total ABAS score provide a good index of adaptive behavior, higher scores corresponding to greater adaptive skills and independency in everyday living. For the sake of the study, those items rated/reported as guessed by parents were scored as zero in each subscale in order to avoid subjective judgments concerning functional changes. In addition, because most individuals in our sample were unemployed, scores in the work skill area were not included in the analyses and not considered when calculating the total ABAS score.

*Early symptoms of dementia*

Dementia Questionnaire for Persons with Intellectual Disability, DMR (Evenhuis HM, Kengen MMF, 2006) Previously named Dementia Questionnaire for persons with Mental retardation. The DMR is a self-reported questionnaire about daily living abilities, which measures specific memory and orientation cognitive skills and social deterioration as a result of dementia and/or severe sensory or psychiatric problems. It consists of 50 items and eight subscales. Combined scores on the first three subscales (Short-term memory, Long-term memory and Orientation) are presented as the Sum of Cognitive Scores (SCS). Combined scores on subscales 4 through to 8 (Speech, Practical skills, Mood, Activity and Interest, and Behavioral disturbance) are presented as the Sum of Social Scores (SOS).

Higher scores in DMR reflect a worse state, while higher punctuations in ABAS-II reflect a better adaptive behavior.

Both questionnaires were given to the caregivers in order to be self- completed while participants completed the neuropsychological testing, to minimize biasing error caused by subjective interviewer effects and to maximize efficiency (information vs time ratio) administration. We ensured they understood how to complete the questionnaires and solved all doubts before and after completion, and checked that all questions were filled.

**A.1 References**

Ball, S. L., Holland, A. J., Treppner, P., Watson, P. C., & Huppert, F. A. (2008). Executive dysfunction and its association with personality and behaviour changes in the development of Alzheimer’s disease in adults with Down syndrome and mild to moderate learning disabilities. *The British Journal of Clinical Psychology / the British Psychological Society*, *47*(Pt 1), 1–29. doi:10.1348/014466507X230967

Benton, A. L., Hamsher, K., & Sivan, A. (1976). *Multilingual aphasia exam*. (U. of I. Press, Ed.). Iowa City: Iowa City: University of Iowa.

Canivez, G. L. (2005). Construct Validity of the Kaufman Brief Intelligence Test, Wechsler Intelligence Scale for Children-Third Edition, and Adjustment Scales for Children and Adolescents. *Journal of Psychoeducational Assessment*, *23*(1), 15–34. doi:10.1177/073428290502300102

Cognition, C. (1996). Cambridge Neuropsychological Test Automated Battery (CANTAB). Cambridge, England: Cambridge Cognition Limited.

Culbertson, C., & Zillmer, E. A. (2005). *Tower of London Drexel University (TOL DX)*. North Tonawanda NY: Multi-Health Systems Inc. (MHS).

de Sola, S., de la Torre, R., Sanchez-Benavides, G., Benejam, B., Cuenca-Royo, A., del Hoyo, L., … TESDAD Study Group, the. (2015). A new cognitive evaluation battery for Down syndrome and its relevance for clinical trials. *Frontiers in Psychology*, *6*. doi:10.3389/fpsyg.2015.00708

Evenhuis HM, Kengen MMF, and E. H. (2006). *Dementia Questionnaire for People with Intellectual Disabilities (DMR).* Amsterdam: Harcourt Test Publishers.

Gerstadt, C. L., Hong, Y. J., & Diamond, A. (1994). The relationship between cognition and action: performance of children 3 1/2-7 years old on a Stroop-like day-night test. *Cognition*, *53*(2), 129–53. Retrieved from http://www.ncbi.nlm.nih.gov/pubmed/7805351

Goldstein, K., Scheerer, M., & Hanfmann, E. (1953). Tests of Abstract and Concrete Thinking. In *Contributions toward medical psychology: Theory and psychodiagnostic methods* (pp. 702–740). New York: Ronald Press Company. Retrieved from http://dx.doi.org/10.1037/11296-008

Harrison, P., & Oakland, T. (2003). *Adaptive Behavior Assessment System (ABAS-II)* (Second Edi.). Los Angeles: PEARSON. Retrieved from http://www.pearsonclinical.com/psychology/products/100000449/adaptive-behavior-assessment-system-second-edition-abas-second-edition.html

Kaufman, A. ., & Kaufman, A. L. (1994). KBIT: Test Breve de Inteligencia de Kaufman. Manual de interpretación. [KBIT: Kaufman’s Brief Intelligence Test. Interpretation Manual]. *Madrid, Spain: TEA*.

Milner, B. (1971). Interhemispheric differences in the localization of psychological processes in man. *British Medical Bulletin*, *27*(3), 272–7. Retrieved from http://europepmc.org/abstract/MED/4937273

Strauss, H., & Lewin, I. (1982). An empirical study of the weigl-goldstein-scheerer color-form test according to a developmental frame of reference. *Journal of Clinical Psychology*, *38*(2), 367–375. doi:10.1002/1097-4679(198204)38:2<367::AID-JCLP2270380226>3.0.CO;2-9

Wechsler, D. (1997). *Wechsler Memory Scale (WMS-III)* (Third Edit.). San Antonio: TX: The Psychological Corporation.
